# Recommendations for progression criteria during external randomised pilot trial design, conduct, analysis and reporting

**DOI:** 10.1186/s40814-023-01291-5

**Published:** 2023-04-15

**Authors:** Katie Mellor, Charlotte Albury, Susan J Dutton, Sandra Eldridge, Sally Hopewell

**Affiliations:** 1 Oxford Clinical Trials Research Unit / Centre for Statistics in Medicine, Nuffield Department of Orthopaedics, Rheumatology and Musculoskeletal Sciences, University of Oxford, Oxford, England; 2 Nuffield Department of Primary Care Health Sciences, University of Oxford, Oxford, UK; 3 Institute of Population Health Sciences, Barts and the London School of Medicine and Dentistry, Queen Mary University of London, London, UK

**Keywords:** Pilot, Feasibility, PAFS, Progression criteria, Recommendations

## Abstract

**Background:**

External randomised pilot trials aim to assess whether a future definitive Randomised Controlled Trial (RCT) is feasible. Prespecified progression criteria help guide the interpretation of pilot trial findings to decide whether, and how, a definitive RCT should be conducted. This commentary presents a set of proposed recommendations for progression criteria to guide researchers when (i) designing, (ii) conducting, (iii) analysing and (iv) reporting external randomised pilot trials.

**Methods:**

Recommendations were developed following a mixed methods approach. This involved (i) a methodological review of pilot trial publications, (ii) a cross-sectional study of pilot trial research funding applications, (iii) qualitative interviews with pilot trial researchers and (iv) a survey of corresponding authors of identified pilot trial publications. Initial recommendations were refined following two consultation stakeholder workshops held in July 2022.

Recommendations for progression criteria for external randomised pilot trials:

i. **Design**: consider progression criteria from the earliest opportunity; map progression criteria to feasibility objectives; consider quantitative and qualitative interpretations of feasibility; provide justification; develop guidelines rather than rules; seek input from relevant stakeholders.

ii. **Conduct**: regularly monitor pilot trial data against progression criteria.

iii. **Analysis**: avoid considering each progression criterion in isolation; engage in discussion with relevant stakeholders; consider context and other factors external to the pilot trial; consider feasibility (can we?) and progression (will we?).

iv. **Reporting**: we propose a reporting checklist in relation to progression criteria and recommend reporting in a table format for clarity.

**Conclusion:**

These recommendations provide a helpful resource for researchers to consider progression criteria at different stages of external randomised pilot trials. We have produced a simple infographic tool to summarise these recommendations for researchers to refer to. Further research is needed to evaluate whether these proposed recommendations should inform future development, or update, of established guidelines for the design, conduct, analysis and reporting of external randomised pilot trials.

## Background

RCTs are integral to the practice of evidence-based medicine [1], however they are often expensive, complicated, and take a long time to set up, conduct, analyse and disseminate findings [2–5]. Despite researchers’ best efforts to deliver gold standard RCTs, many RCTs do not adequately answer their research question [6, 7], resulting in research waste and inefficiencies.

Feasibility studies ask: can the future definitive trial be done, should we proceed with it, and if so, how? [8] Pilot trials are a type of feasibility study. They ask the same question, but also ‘pilot’ or conduct all or part of a future trial on a smaller scale [8]. External pilot trials are small stand-alone studies, where any outcome data that is collected does not contribute to the definitive trial analysis [8, 9]. This is the key difference from an internal pilot trial which is embedded within a definitive RCT forming the first phase, therefore any data collected does contribute to the definitive trial analysis [9, 10].

Progression criteria help researchers interpret their pilot trial findings to decide whether, and how, to proceed with a future definitive trial. It is important that progression criteria are specified before the pilot trial begins (a priori) to avoid introducing bias associated with establishing progression criteria once external pilot trial findings are known. If progression criteria are not set a priori, there is a risk that pilot trials may be optimistic in reporting that a future RCT is feasible [11] and progress to a definitive RCT without modification or acknowledgement of potential limitations [12].

Although progression criteria are now required by some research funders in pilot trial funding applications [13] and are specified as a reporting item in the CONSORT extension for external Pilot and Feasibility Studies (PAFS) [14], existing recommendations are largely based on individual case studies or have not been developed specifically with external randomised pilot trials in mind.

Using a mixed methods approach, we conducted four distinct but complimentary research studies to examine how progression criteria inform assessment of external randomised pilot trial feasibility. In this commentary we present our recommendations for best practice.

## Development of recommendations

The four research studies that underpin these recommendations are reported in detail, or are currently under review, elsewhere. In study one, we examined the current reporting of progression criteria in a large representative sample of external randomised pilot trial protocol and result publications (published between January 2018 and December 2019) [15]. In study two, we examined the inclusion of progression criteria in research funding applications by conducting a cross-sectional study of progression criteria proposed in external randomised pilot trial funding applications submitted to NIHR Research for Patient Benefit (RfPB) (with a funding decision between July 2017 and July 2019) [16]. In study three, we explored pilot trial team member perceptions and experiences of developing and using progression criteria in practice by conducting a qualitative research study with key stakeholders (between December 2020 and July 2021) [17]. Finally, in study four, we examined whether identified pilot trials were considered feasible, and progressed to further research, by conducting a survey study (between January 2022 and February 2022) of corresponding authors of publications included in study one [18].

These four studies were conducted over a three-year period between 2019 and 2022. The findings were iteratively triangulated to identify areas where recommendations about best practice for progression criteria might be useful. The lead author (KM) drafted initial recommendations which were then refined by the wider study team and following feedback received from two external stakeholder meetings (July 2022). We wanted to capture a range of different perspectives on our initial recommendations from stakeholders with different trial roles. Stakeholders were invited following contribution, participation or engagement in earlier research [15–18] or were suggested by members of a wider Pilot and Feasibility Studies Working Group. External stakeholders included three trial investigators, four trial statisticians, six trial methodologists/health services researchers, one research funder manager and one professor of primary care pharmacy.

**Fig. 1:**
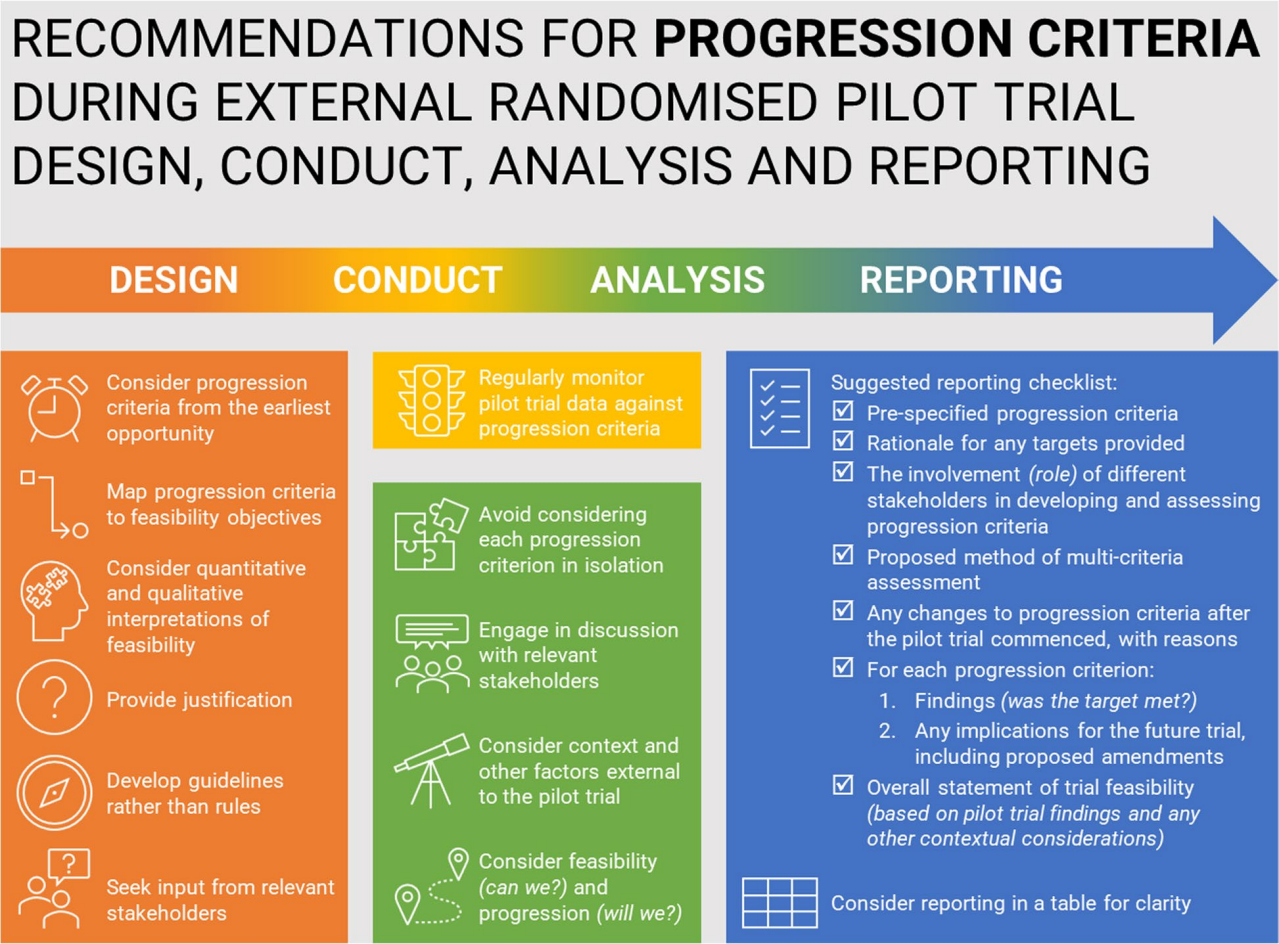
Infographic to summarise recommendations for progression criteria

The final recommendations are structured to indicate considerations for progression criteria when (i) designing, (ii) conducting, (iii) analysing and (iv) reporting external randomised pilot trials. In addition an infographic tool has been produced to provide a helpful summary (See Fig. 1).

## Recommendations for progression criteria

### Recommendations for progression criteria during pilot trial design

#### Consider progression criteria from the earliest opportunity

Researchers should consider progression criteria from the onset when designing their pilot trial, and some research funders now require that progression criteria are included in pilot trial funding applications [13]. Early consideration of progression criteria provides a valuable opportunity for researchers to think about where their uncertainties lie and what problems that they might face, potentially saving time and effort in the long run.

#### Map progression criteria to feasibility objectives

Researchers should consider whether progression criteria are needed for each of their feasibility objectives. Too often progression criteria only focus on recruitment and retention and do not account for other feasibility issues that might also be pertinent to the success of the definitive RCT. For example, if researchers are conducting qualitative research as part of their pilot trial, they should consider whether, and how, these findings will inform progression criteria. Not all data collected during the external pilot trial needs to inform progression criteria, some might be collected to pilot and refine trial processes without contributing to progression decision making, for example to optimise efficiency of recruitment processes, training or intervention delivery. However, mapping progression criteria to feasibility objectives as appropriate can ensure that they are developed with the definitive trial in mind. To contextualise progression criteria researchers might also find it useful to associate criteria with specific timeframes or settings e.g. monthly or site-specific targets.

#### Consider quantitative and qualitative interpretations of feasibility

Although it has become standard practice to use numerical targets for progression criteria, researchers should be mindful that this might not always be appropriate. Although quantifiable targets might seemingly ensure transparent progression criteria assessment, they should be avoided where they are not meaningful.

**Fig. 2:**
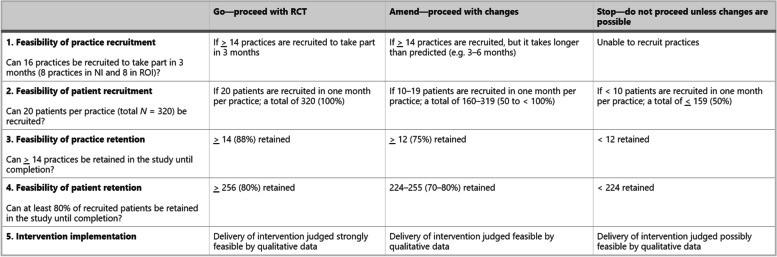
Example progression criteria including both quantitative and qualitative data. Reproduced from Hynes et al (2022) Pilot Feasibility Stud 8:1–16, published CC-BY 4.0

Mixed method approaches to PAFS data collection have been recommended [19, 20]. Guidance for maximising qualitative research in feasibility studies, outlining feasibility questions that might be best addressed using qualitative research methods, approaches to qualitative data collection and analysis have previously been outlined [21]. Researchers might therefore opt to have a combination of numerical and non-numerical progression criteria where they have used qualitative research methods as part of their external pilot trial. An example is presented in Fig. 2 [22].

Not every pilot trial will have a formally embedded qualitative research study e.g. a process evaluation. However, this does not negate the importance of considering other ‘soft intelligence’ gained from conducting the pilot trial which might be interpretated qualitatively, for example through ‘informal conversations’ with healthcare professionals at investigative sites [23]. These informal conversations can occur anywhere at any time and are likely already widely being used by research teams to generate new ideas about intervention or trial deliverability that are investigated by changing aspects of the pilot trial design. However, new strategies are needed to ensure that any data generated through informal conversations that informs and underpins changes to trial design does not go undocumented. One example is to maintain a ‘lessons learned’ document to capture problems faced during the pilot trial, any attempts to resolve these issues, and whether they worked or not. Bugge et al suggest that solutions to problems faced during the pilot trial might be identified through literature searching; debate/ brainstorming within the research team; and, if available, through analysis of existing feasibility study data [24].

#### Provide justification

Researchers should provide some justification for any progression criteria including any stated numerical progression criteria targets to indicate how they were derived. Rationale need not be statistical as pilot trials are usually underpowered for hypothesis testing, but some rationale for criteria should be given (e.g. based on feasibility objectives; clinical or contextual assumptions; pragmatically derived; based on previous feasibility or observational work; developed using consensus methods involving a range of stakeholders). Investigators should be aware that small pilot trial sample sizes might mean that any estimates of rates are subject to considerable uncertainty [25].

#### Develop guidelines rather than rules

Progression criteria are best viewed as guidelines rather than strict rules. Researchers should therefore develop progression criteria that will help identify and explore potential challenges with their trial design to inform the development of actionable solutions.

For example, it is becoming increasingly common for investigators to use a traffic light or Red Amber Green (RAG) approach for progression criteria. This approach is also recommended and widely used in RCTs with internal pilot trial phases [26, 27]. There are no agreed hard and fast rules for the meanings attributed to each colour, but typically measures below a lower (red) threshold have indicated that the pilot trial is not feasible [stop], above a higher (green) threshold that it is feasible [go], and between the two (amber) that it might be feasible if appropriate changes can be made [amend] [14]. However, for many external pilot trials a red criterion might not necessarily mean that the definitive trial is not feasible. Instead, it might be more appropriate to think of the RAG system as a way to highlight, and draw attention to, problems that have been faced in the pilot trial, with red indicating major problems that require urgent attention (and perhaps cannot be remedied), amber indicating minor problems that require attention, and green indicating areas of no concern.

#### Seek input from relevant stakeholders

Researchers should try to involve a broad range of stakeholders to develop, or agree, progression criteria so that targets are more meaningful and less prone to bias. A multidisciplinary approach to progression criteria development might involve consulting a Research Design Service (RDS) or Clinical Trials Unit (CTU). Seeking input from clinical colleagues who are implementing the pilot trial can help ensure that progression criteria make sense for different clinical contexts, recognising that pilot trial sites are not always reflective of the sites used in the main trial. Researchers might also want to involve Patient and Public Involvement (PPI) representatives to agree their progression criteria, and if applicable, agree progression criteria with their Trial Steering Committee (TSC) in advance, as is recommended for internal pilot trials [26]. Young et al have described using plain English explanations and a useful everyday analogy to facilitate understanding of progression criteria among patient and clinician groups when co-producing progression criteria [28]. Finally, researchers should also consider appropriate funding sources for their future definitive trial if they are able to demonstrate feasibility and ensure that progression criteria encompass any recommendations for feasibility assessment made by the intended definitive trial funder.

### Recommendations for progression criteria during pilot trial conduct

#### Regularly monitor pilot trial data against progression criteria

Researchers might find it useful to revisit their progression criteria targets regularly throughout their pilot trial at important trial milestones, or as a standing agenda item at multidisciplinary team meetings e.g. the Trial Management Group (TMG) and/or TSC. This might be particularly important when any changes to the pilot trial design are made, so that researchers can determine whether these changes have improved the trial design (i.e. whether indicators of feasibility are improving or trending towards green progression criteria). Criteria that fall within the ‘red’ domain will signify where urgent attention is needed to identify, outline and pilot actionable solutions in response to problems that are being faced.

There might be instances where changes to the pilot trial design might directly or indirectly affect the applicability of the progression criteria. In these instances, researchers might re-define their progression criteria, following consultation with relevant stakeholders (e.g. those who initially input into their development and any newly identified stakeholders of relevance), to ensure that they are still usable indicators of trial feasibility. Where any changes to progression criteria are made, reasons for these changes should be fully reported in pilot trial publications. Note that unlike an internal pilot trial, any outcome data collected during an external pilot trial does not contribute to the definitive trial analysis [8].

### Recommendations for progression criteria during pilot trial analysis

#### Avoid considering each progression criterion in isolation

Most pilot trials will have multiple progression criteria, so researchers should consider their method of multi-criteria assessment. Researchers might opt to take a holistic approach to feasibility assessment and consider criteria in relation to each other, the implications for the future definitive trial, and whether any solutions to poorly performing criteria have been identified to come to an overall conclusion of feasibility. This may or may not involve weighting specific criteria that are regarded as more fundamental than others. Outlining clear, actionable solutions where progression criteria are not met (e.g. are within red or amber ranges) is an important component of feasibility assessment that should not be overlooked. This is in line with recommendations for evaluating the feasibility of internal pilot trials which suggests that definitive trial funders acknowledge the importance of considering supplementary data e.g. a ‘rescue plan’ that outlines any problems encountered and how they were addressed [26]. An alternative approach suggested for multi-criteria assessment is to determine overall progression based on the worst-performing criterion (e.g. if one criterion is not met then the trial is not feasible) [29].

#### Engage in discussion with relevant stakeholders

Just as it is important to engage a broad range of stakeholders in developing progression criteria, it is important to engage different stakeholders at the end of the pilot trial when determining feasibility. At this stage it might be particularly useful to speak to people outside of the immediate trial team, e.g. clinical and healthcare professionals who were implementing the intervention or trial processes, to gain a comprehensive understanding about what might have worked well and what might require improvement in the definitive trial. For example, a behavioural science approach might be used to identify specific challenges and potential solutions [30].

#### Consider context and other factors external to the pilot trial

Since progression criteria are developed during the early pilot trial design stage, they do not account for unforeseen events and challenges researchers might face whilst conducting the pilot trial. Researchers should account for these factors, drawing on evidence and experience generated from doing the pilot trial, and any relevant external evidence generated outside of their pilot trial, when drawing conclusions about feasibility and progression.

#### Consider feasibility (can we?) and progression (will we?)

Researchers should recognise that feasibility assessment (can we do it?) and progression decision making (will we do it?) are complimentary but distinct considerations. There might be instances where pilot trials are considered feasible (i.e. based on progression criteria and assessment of deliverability) but do not progress for other reasons that are external to the pilot trial design (e.g. funding might not be available, the healthcare context might have changed, the intervention might now be superseded, or the CI might not intend to pursue the definitive trial at this time). It is important to be transparent about both whether the pilot trial is considered feasible, and whether researchers intend to progress to further research. Doing so might further evidence wider challenges with pilot trial progression for the research community to address, or even present the opportunity for other research groups to advance completed pilot trials that were feasible but might not otherwise progress.

### Recommendations for progression criteria reporting

#### Suggestions for what information to report

Clear and transparent reporting of progression criteria ensures that trial feasibility is assessed with integrity and rigour. Clear reporting also enhances the wider usability of pilot trial findings. For example researchers can adequately determine whether progression criteria for one pilot trial might be applicable to another if information about how those criteria were developed is provided. This should encourage better research practice and avoid researchers providing generic progression criteria that are not meaningful.

To improve transparency of progression criteria reporting, we propose that the information detailed in Table 1 is included in external randomised pilot trial publications. The suggested items for protocol publications might also be applicable to researchers who are developing funding applications for external randomised pilot trials.

**Table 1:** Suggested reporting items and rationale

**Suggested item**	**Rationale**	**Protocol**	**Result**
**Methods**			
Pre-specified progression criteria	To ensure the pilot trial meets its (feasibility) objectives, specific measures or assessments should be defined to address each separate objective ^a^. A range of methods can be used, and are often based on quantitative descriptive statistics but might also include qualitative narrative descriptions ^b^. It is these feasibility objectives and measures that progression criteria should be based on. Estimates of rates in pilot trials may be subject to considerable uncertainty, so investigators should be cautious about setting definitive thresholds that could be missed simply due to chance variation. Instead it is becoming increasingly common to use a traffic light system for criteria used to judge feasibility, whereby measures (e.g. recruitment rates) below a lower (red) threshold indicate major problems that require urgent attention (and perhaps cannot be remedied), amber indicating minor problems that require attention, and green indicating areas of no concern ^c^.	✓	✓
Rationale for progression criteria, including any data or clinical assumptions supporting any targets provided	It should be stated how progression criteria were derived to ensure transparent feasibility assessment. Rationale need not be statistical as pilot trials are usually underpowered for hypothesis testing, but some rationale for criteria should be given (e.g. based on feasibility objectives; clinical or contextual assumptions; pragmatically derived; based on previous feasibility or observational work; developed using consensus methods).	✓	*
Brief description of the involvement (role) of different stakeholders in developing, or agreeing, progression criteria	A multidisciplinary team of stakeholders may be involved in developing, or agreeing, progression criteria to avoid bias. Stakeholders who might contribute to progression criteria development include: Clinical Trials Unit; Research Design Service; clinical professionals who will be implementing the pilot trial; PPI representatives; research funders; an independent Trial Steering Committee.	✓	*
Proposed method of multi-criteria assessment	Where pilot trials have multiple progression criteria, researchers should indicate a method of multi-criteria assessment. Researchers might take a holistic approach and consider whether targets were met and whether solutions to poorly performing criteria have been identified to come to an overall conclusion of feasibility. This may involve weighting of specific criteria that are regarded as more fundamental than others. Alternatively, researchers might opt to take a more structured approach to multi-criteria assessment and determine overall progression based on the worst-performing criterion (e.g. if one criterion is not met then the trial is not feasible).	✓	*
**Results**			
Any changes to progression criteria after the pilot trial commenced, with reasons	An assessment or measure might change during a pilot trial because the change enables investigators to glean more information about the operation of the intervention or for reasons of acceptability or practicability ^d^. These changes might directly or indirectly impact on the applicability of the pre-specified progression criteria. Because of the usefulness of such information to the overall assessment of trial feasibility, all changes to progression criteria should be reported.		✓
For each progression criterion, findings in terms of whether criteria indicate feasibility and if applicable, whether numerical targets were met	Findings for each progression criteria should be provided to demonstrate which aspects of the pilot trial were considered feasible. Where a RAG system was used researchers should indicate whether each progression criterion was above the upper threshold (green), below the lower threshold (red), or between the two (amber). For any progression criteria that are not based on numerical targets, a clear statement to reflect whether the findings indicate feasibility is sufficient.		✓
For each progression criterion, implications for the future definitive trial, including any proposed amendments	It is important to understand how the pilot trial findings have informed the definitive RCT ^e^. For each progression criterion researchers should state any implications that their findings have for the definitive trial design. Any proposed changes to trial design should be based on evidence, or experience, generated from the pilot trial.		✓
Overall statement of trial feasibility (based on pilot trial findings and any other contextual considerations)	Researchers should provide an overall statement of feasibility based on pilot trial findings and any other contextual considerations, or external evidence, generated whilst conducting the pilot trial. This is important because sometimes progression criteria do not adequately reflect trial feasibility. For example, a pilot trial might be feasible (based on assessment of progression criteria) but not progress for other reasons such as unanticipated challenges or factors external to the pilot trial.		✓

^*^Items might be omitted from pilot trial results publications if the pilot trial protocol is published and referenced

^a^CONSORT 2010 statement: extension to randomised pilot and feasibility trials, Item 6a

^b^CONSORT 2010 statement: extension to randomised pilot and feasibility trials, Item 12a

^c^CONSORT 2010 statement: extension to randomised pilot and feasibility trials, Item 6c

^d^CONSORT 2010 statement: extension to randomised pilot and feasibility trials, Item 6b

^e^CONSORT 2010 statement: extension to randomised pilot and feasibility trials, Item 22

#### Consider reporting in a table for clarity

**Fig. 3:**
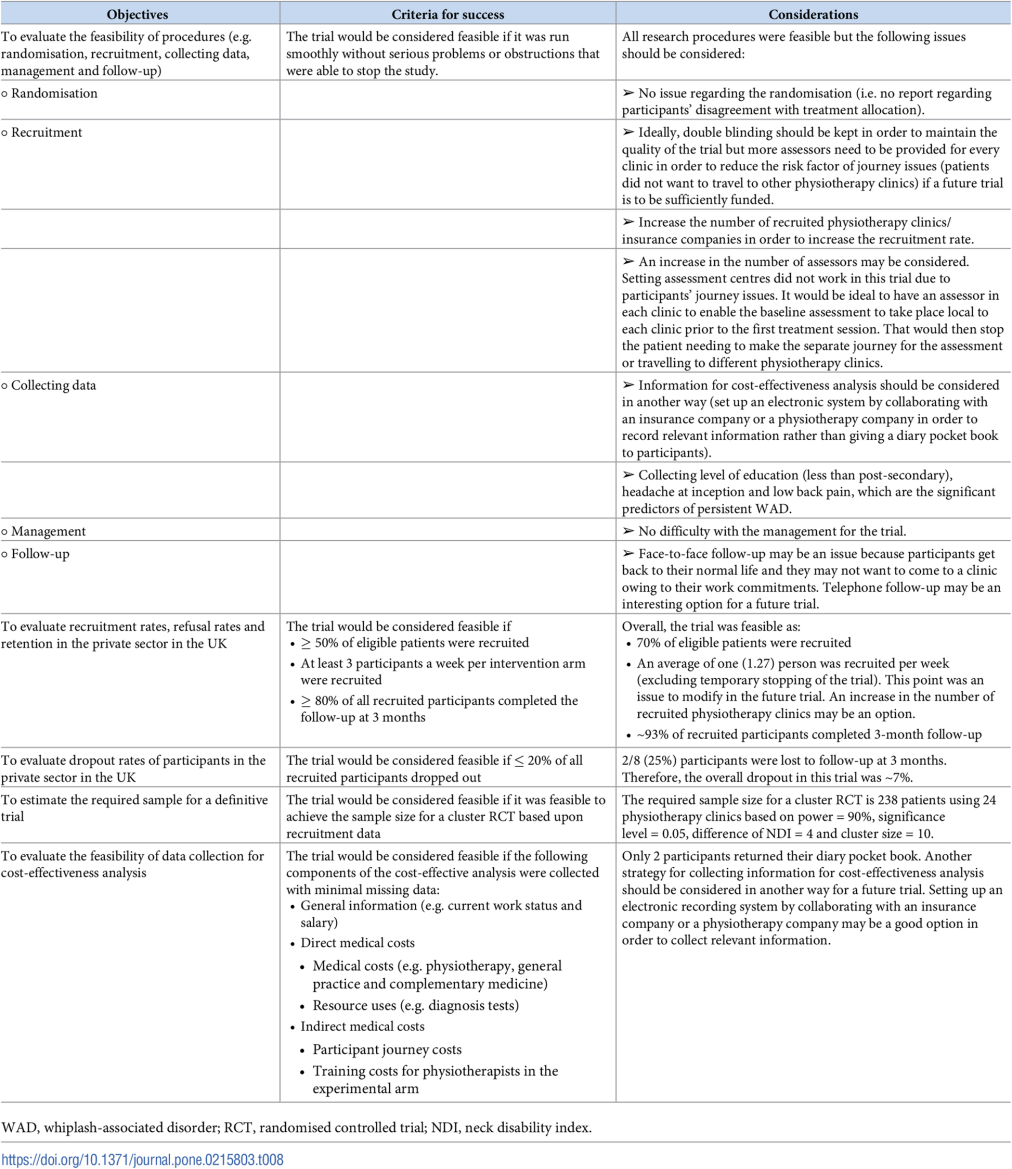
Example table to report progression criteria findings and implications for definitive RCT. Reproduced from Wiangkham et al (2019) PLoS One 14:e0215803, published CC-BY 4.0

To promote clarity researchers should consider reporting progression criteria and their associated findings in a table format. This is particularly useful for completed trials to report the progression criteria set, the corresponding finding and the implications for the future RCT design. GRADE (Grading of Recommendations, Assessment, Development and Evaluation) is a transparent approach to grading the quality of evidence to inform healthcare recommendations. GRADE recommends using Summary of Findings (SoF) tables to provide a concise summary of key information that underpins recommendations. This format of evidence synthesis and reporting strikes a balance between simplicity of information presentation and completeness (or transparency) [31], and some pilot trial publications have effectively reported pilot trial findings in a similar format [32–35]. One example is presented in Fig. 3 [35].

## Conclusions

Progression criteria for external randomised pilot trials are becoming increasingly required in publications and funding applications; however it is unclear how they should be developed, assessed and reported. To our knowledge, these are the first recommendations developed for progression criteria across key stages of external randomised pilot trials. Although these recommendations were developed with external randomised pilot trials in mind, it is possible that they might also apply to non-randomised or single-arm pilot or feasibility studies that pre-specify progression criteria to inform assessment of trial feasibility. We hope that these practical recommendations will support researchers, funders, editors and peer reviewers to include and encourage the inclusion of clear and useful progression criteria in pilot trials, leading to improved feasibility assessment and reduced research waste. Further research is needed to evaluate whether these proposed recommendations should inform future development, or update, of established guidelines for the design, conduct, analysis and reporting of external randomised pilot trials.

## Data Availability

Data will be made available upon request and included in a DPhil thesis published open access through the Oxford University Archive upon KM’s DPhil completion.

## Abbreviations

ADePT: A process for Decision-making after Pilot and feasibility Trials
CI: Chief Investigator
CONSORT: Consolidated Standards of Reporting Trials
CReDECI: Criteria for Reporting the Development and Evaluation of Complex Interventions in healthcare
CTU: Clinical Trials Unit
PAFS: Pilot and Feasibility Studies
PPI: Patient and Public Involvement
RAG: Red Amber Green
RCT: Randomised Controlled Trial
RDS: Research Design Service
RfPB: Research for Patient Benefit
TMG: Trial Management Group
TSC: Trial Steering Committee
